# Association of Laminar Airflow During Primary Total Joint Arthroplasty With Periprosthetic Joint Infection

**DOI:** 10.1001/jamanetworkopen.2020.21194

**Published:** 2020-10-16

**Authors:** Qiaojie Wang, Chi Xu, Karan Goswami, Timothy L. Tan, Javad Parvizi

**Affiliations:** 1 Rothman Orthopaedic Institute at Thomas Jefferson University, Philadelphia, Pennsylvania; 2 Department of Orthopaedic Surgery, Shanghai Jiaotong University Affiliated Shanghai Sixth People’s Hospital, Shanghai, China; 3 Department of Orthopaedic Surgery, General Hospital of People’s Liberation Army, Beijing, China

## Abstract

**Importance:**

Whether laminar airflow (LAF) in the operating room is effective for decreasing periprosthetic joint infection (PJI) after total joint arthroplasty remains a clinically important yet controversial issue.

**Objective:**

To investigate the association between operating room LAF ventilation systems and the risk of PJI in patients undergoing total joint arthroplasty.

**Design, Setting, and Participants:**

A retrospective cohort study was conducted of 6972 consecutive patients undergoing primary total knee arthroplasty or total hip arthroplasty at 2 surgical facilities within a single institution from January 1, 2013, to September 15, 2017, with a minimum of 1 year of follow-up. All procedures were performed by 5 board-certified arthroplasty surgeons. Statistical analysis was performed from January 1, 2014, to September 15, 2018.

**Exposure:**

Patients underwent total joint arthroplasty in operating rooms equipped with either LAF or turbulent airflow.

**Main Outcomes and Measures:**

Patient characteristics were extracted from clinical records. Periprosthetic joint infection was defined according to Musculoskeletal Infection Society criteria within 1 year of the index arthroplasty. A multivariable logistic regression model was performed to explore the potential association between LAF and risk of PJI at 1 year, and then a sensitivity analysis using propensity score matching was performed to further validate the findings.

**Results:**

A total of 6972 patients (2797 who underwent total knee arthroplasty and 4175 who underwent total hip arthroplasty; 3690 women [52.9%]; mean [SD] age, 63.9 [10.7] years) were included. The incidence of PJI within 1 year for patients from the facility without LAF was similar to that of patients from the facility with LAF (0.4% [12 of 3027] vs 0.5% [21 of 3945]). In the multivariable logistic regression analysis, after all confounding factors were taken into account, the use of LAF was not associated with a reduction of the risk of PJI (adjusted odds ratio, 0.94; 95% CI, 0.40-2.19; *P* = .89). After propensity score matching, there was no significant difference in the incidence of PJI within 1 year for patients who underwent total joint arthroplasty at these 2 sites.

**Conclusions and Relevance:**

This study suggests that the use of LAF in the operating room was not associated with a reduced incidence of PJI after primary total joint arthroplasty. With an appropriate perioperative protocol for infection prevention, LAF does not seem to play a protective role in PJI prevention.

## Key Points

**Question:**

Is the use of laminar airflow during total joint arthroplasty procedures associated with reduced risk of subsequent periprosthetic joint infection?

**Findings:**

In this cohort study of 6972 patients, the rate of periprosthetic joint infection was similar in procedures performed in operating rooms with or without laminar airflow (0.5% vs 0.4%).

**Meaning:**

This study suggests that laminar airflow may not play a role in prevention of periprosthetic joint infection.

## Teaser

This cohort study examines the association between operating room laminar airflow ventilation systems and the risk of periprosthetic joint infection in patients undergoing total joint arthroplasty.

## Introduction

The economic burden to manage periprosthetic joint infection (PJI) continues to increase in the United States and is expected to exceed $1.6 billion by 2020.^1^ Thus, prevention of PJI after total joint arthroplasty (TJA) is important. Many strategies are known to be effective in reducing the incidence of PJI.^2,3,4,5,6^ One such strategy, based on teachings of Sir John Charnley,^7^ is associated with the use of an ultraclean operating room. As ambient air in the operating room is believed to be a route of microbial entry into an open clean surgical wound, control of the operating room environment via an appropriate ventilation system is critical for the prevention of PJI.^8,9,10^

Although a few previous studies have demonstrated that laminar airflow (LAF) ventilation is associated with significantly decreased air microbial contamination vs conventional turbulent ventilation systems,^11,12,13^ the effectiveness of LAF in decreasing infection after TJA has been a subject of controversy in recent decades. A meta-analysis by Bischoff et al^14^ suggested that there is no benefit associated with using LAF compared with conventional turbulent ventilation for reducing the risk of surgical site infection (SSI) after arthroplasty of the hip and the knee. Moreover, several studies using data from national joint registries have concluded that the use of LAF could increase the infection rate after TJA.^15,16^ These studies have led the World Health Organization to a conditional recommendation that LAF should not be used to reduce the risk of SSI for patients undergoing TJA.^17^

However, most published reports on PJI in the literature have been obtained from national surveillance systems and registries, which may underestimate the percentage of PJI by up to 40%.^18^ Meanwhile, these data were submitted by numerous hospitals, raising the possibility of significant differences in hospital or surgeon volume, patient characteristics, or implementation of other SSI prevention measures, which may confound the results. Furthermore, previous studies used SSI as their primary outcome, which was typically not a well-defined or standardized end point. A well-designed study with an adequate sample size and granular data from a single institution is thus lacking, and such data may potentially provide more reliable and clinically relevant information when investigating the association of LAF with the risk of PJI.

We sought to use the unique structure of our institution where several high-volume surgeons perform TJA at 2 sites with the same perioperative SSI and PJI prevention measures but different operating room ventilation systems. The operating room environment at the first site uses a vertical LAF system with a high-efficiency particulate air (HEPA) filter, and the second site uses a conventional turbulent ventilation system with HEPA-filtered air in the operating room. The aim of the present study was to evaluate the association of operating room ventilation systems with PJI rates after TJA, after matching for patient characteristics and other potential confounding factors.

## Methods

### Patients

We performed a retrospective cohort study of consecutive patients undergoing primary total knee arthroplasty and total hip arthroplasty between January 1, 2013, and September 15, 2017, at 2 surgical facilities (center A and center B) within a single institution. All the procedures were performed by 5 high-volume, fellowship-trained, arthroplasty surgeons (including J.P.) who perform TJA in both facilities as part of their routine practices. Patients were allocated by their surgeons to one of the facilities to undergo TJA based on each patient’s preference and their overall risk of perioperative complications. We included unilateral primary total knee arthroplasty and total hip arthroplasty with available detailed data and with a minimum follow-up of 1 year. Exclusion criteria included the following: simultaneous bilateral arthroplasty, conversion arthroplasty (with a history of prior surgery on the index joint with or without retained hardware), revision arthroplasty, and simultaneous multiple site surgical procedures. Patient characteristics, such as age, sex, body mass index (BMI), Charlson Comorbidity Index (CCI), current smoking status, total operative time from the first incision to closure, operating surgeon, and length of hospital stay, were identified from institutional records. This study was conducted after approval from the Thomas Jefferson University Institutional Review Board and written informed consent was obtained from all study participants. This study followed the Strengthening the Reporting of Observational Studies in Epidemiology (STROBE) reporting guideline for cohort studies.

### Ventilation Systems in the 2 Different Operating Room Environments

The ventilation system at center A uses LAF with HEPA filters (Medical Air Technology), with a minimum differential pressure of 0.030 cm (0.012 in) of water column. It uses recirculating air, with a mean of 19.18 air exchanges per hour. It meets both National Institute of Occupational Safety and Health (NIOSH) and American Society of Heating, Refrigerating and Air-Conditioning Engineers (ASHRAE) standards but not the Centers for Disease Control and Prevention (CDC) standard. The system is maintained once every 2 years. Conventional turbulent ventilation with HEPA-filtered air was used at center B, with a minimum differential pressure of 0.030 cm (0.020 in) of water column. It uses recirculating 20% outside air with a mean of 37 air exchanges per hour. It meets both the CDC and ASHRAE standards but not the NIOSH standard. The system is maintained once every year. The area and height of the operating rooms are similar between the 2 facilities. All surgical personnel wear surgical body suits (“space suits”) during the procedures. Forced-air warming systems were routinely used for the patients at both facilities during the study period.

### Perioperative Measures for Infection Prevention

The same standard for sterilization of surgical instruments was used in both facilities. Similar perioperative measures for infection prevention were also implemented at both facilities during the study period, in keeping with the overall institutional protocol. These measures included the same protocol for perioperative antibiotic prophylaxis, the use of surgical irrigation with dilute povidone-iodine, the use of antibiotic-loaded cement for cemented prostheses, and the application of Aquacel Ag hydrofiber dressing (ConvaTec) for every patient. Both sites also used the same protocol for venous thromboembolism prophylaxis and postoperative rehabilitation.

### Outcome Measures

Our primary outcome measure was PJI within 1 year of the index arthroplasty. All potential postoperative SSIs were identified with extensive review of our institutional databases, including records of hospital readmissions and postdischarge procedures (*Current Procedural Terminology* codes) and patient diagnoses (*International Classification of Diseases, Ninth Revision* codes). Each potential infection was then reviewed manually and the final diagnosis of PJI was based on the Musculoskeletal Infection Society criteria.^19^

### Statistical Analysis

Statistical analysis was performed from January 1, 2014, to September 15, 2018. The χ^2^ test was applied for categorical variables (sex, joint, and the occurrence of infection) to evaluate significant differences between groups and an independent 2-sample *t* test or Mann-Whitney test was used for continuous variables (CCI, operative time, length of stay, BMI, and age). Univariate logistic regression was performed to investigate the association between operating room ventilation systems and PJI. In the multivariable logistic regression model, we adjusted for all variables listed in Table 1, including age, sex, BMI, CCI, smoking status, joint type (hip or knee), operative time group (<90 minutes or not), and length of hospital stay group (<2 days or not). Quantitative variables were modeled linearly. Statistical interactions and regression diagnostics were not examined. Unadjusted and adjusted odds ratios (ORs) with 95% CIs were calculated.

**Table 1.** Basic Characteristics of Patients Included in the Study Before Propensity Score Matching

Variables	Patients, No. (%)		*P* value
	Center A (with LAF) (n = 3945)	Center B (without LAF) (n = 3027)	
Age, mean (SD), y	65.0 (10.7)	62.3 (10.5)	<.001
BMI, mean (SD)	29.4 (5.1)	29.3 (4.8)	.43
CCI score, mean (SD)	2.7 (1.6)	2.9 (1.2)	<.001
Sex			
Female	2103 (53.3)	1587 (52.4)	.47
Male	1842 (46.7)	1440 (47.6)	
Smoker			
No	3688 (93.5)	2902 (95.9)	<.001
Yes	257 (6.5)	125 (4.1)	
Joint			
Hip	2330 (59.1)	1845 (61.0)	.11
Knee	1615 (40.9)	1182 (39.0)	
Operative time, mean (SD), min	68.7 (19.3)	65.1 (20.4)	<.001
Operative time group, min			
<90	3491 (88.5)	2640 (87.2)	.11
≥90	454 (11.5)	387 (12.8)	
Length of hospital stay, mean (SD), d	1.65 (1.1)	1.20 (0.5)	<.001
Length of hospital stay group, d			
>2	593 (15.0)	80 (2.6)	<.001
≤2	3352 (85.0)	2947 (97.4)	
PJI within 1 y of index arthroplasty			
No	3924 (99.5)	3015 (99.6)	.41
Yes	21 (0.5)	12 (0.4)	

Abbreviations: BMI, body mass index (calculated as weight in kilograms divided by height in meters squared); CCI, Charlson Comorbidity Index; LAF, laminar airflow; PJI, periprosthetic joint infection.

To verify our results further, a sensitivity analysis was performed using 1:1 propensity score matching (PSM) based on patient’s age, sex, BMI, smoking status, CCI, operative time (<90 minutes or not), length of hospital stay (<2 days or not), joint (hip or knee), surgeon, and the year of the index surgery. The nearest-neighbor matching method was used, and the maximum difference between propensity probabilities for matching was set at 0.05. A standardized mean difference for each covariate was examined after applying the PSM adjustment. Standardized mean differences of 10% or less were considered suggestive of covariate balance.^20^ After matching, patient characteristics were compared again between the 2 groups. In the PSM subsample, univariate logistic regression analysis was performed to estimate matched ORs of PJI. *P* < .05 was considered significant. All of the statistical analyses were performed with the statistical software packages R, version 3.1 (R Foundation for Statistical Computing)^21^ and EmpowerStats (X&Y Solution Inc).^22^

## Results

A total of 6972 patients (2797 total knee arthroplasty and 4175 total hip arthroplasty; 3690 women [52.9%]; mean [SD] age, 63.9 [10.7] years) were included. Of these, 3027 patients underwent TJA in the facility without LAF (center B) and 3945 patients underwent TJA in the facility with LAF (center A). Basic characteristics of these patients before PSM are shown in Table 1. There were 21 PJI cases within 1 year of the index arthroplasty among patients from center A and 12 PJI cases among patients from center B. The incidence of PJI within 1 year for patients from center B (0.4%; 95% CI, 0.2%-0.6%) was not statistically significantly different from that of patients from center A (0.5%; 95% CI, 0.3%-0.8%) (*P* = .41).

Univariate logistic regression analysis revealed that the use of LAF was not statistically significantly associated with PJI within 1 year, with an OR of 1.34 (95% CI, 0.66-2.74; *P* = .41). In the multivariable logistic regression analysis, after all confounding factors (including age, sex, BMI, CCI, current smoking status, operative time, operating surgeon, and length of hospital stay) were taken into account, the use of LAF in the operating room was not significantly associated with a reduction of the risk of PJI (adjusted OR, 0.94; 95% CI, 0.40-2.19; *P* = .89).

In the sensitivity analysis using 1:1 PSM, we generated a subsample of 2234 patients from center A and 2234 matched patients from center B (Table 2). The quality of PSM was considered balanced (all standardized mean differences <10%). The incidence of PJI within 1 year for patients in the matched subsample was 0.4% (n = 9) from center A and 0.5% (n = 11) from center B, with no statistically significant difference between the groups (*P* = .60). In the univariate analysis, the use of LAF was not associated with the rate of PJI within 1 year (PSM OR, 1.24; 95% CI, 0.55-2.82; *P* = .60).

**Table 2.** Basic Characteristics of Patients After Propensity Score Matching

Variables	Patients, No. (%)		SMD	*P* value
	Center A (with LAF) (n = 2234)	Center B (without LAF) (n = 2234)		
Age, mean (SD), y	63.1 (10.9)	62.8 (10.1)	0.0306	.31
BMI, mean (SD)	29.2 (5.3)	29.3 (4.8)	0.0130	.67
CCI score, mean (SD)	2.8 (1.8)	2.9 (1.1)	0.0153	.61
Sex				
Female	1190 (53.3)	1166 (52.2)	0.0215	.49
Male	1044 (46.7)	1068 (47.8)		
Smoker				
No	2146 (96.1)	2135 (95.6)	0.0246	.46
Yes	88 (3.9)	99 (4.4)		
Operative time group, min				
<90	1951 (87.3)	1945 (87.1)	0.0080	.82
≥90	283 (12.7)	289 (12.9)		
Length of hospital stay, d				
>2	87 (3.9)	79 (3.5)	0.0189	.58
≤2	2147 (96.1)	2155 (96.5)		
Joint				
Hip	1388 (62.1)	1367 (61.2)	0.0193	.54
Knee	846 (37.9)	867 (38.8)		

Abbreviations: BMI, body mass index (calculated as weight in kilograms divided by height in meters squared); CCI, Charlson Comorbidity Index; LAF, laminar airflow; SMD, standardized mean difference.

## Discussion

Although some studies have revealed that operating rooms equipped with LAF can greatly reduce levels of particles and bacteria in the air compared with conventional turbulent systems,^23^ the clinical association of LAF with the risk of PJI remains controversial. In the present study, we evaluated the association of operating room ventilation systems with PJI rates after TJA, using PSM data from patients who underwent TJA performed by the same group of surgeons at a single institution and were treated with the same perioperative protocols for infection prevention, except for different operating room ventilation systems. The results of this study, the first of its kind in terms of design to our knowledge, revealed that TJA performed in operating rooms with LAF has similar rates of PJI as that of operating rooms with conventional turbulent ventilation. Laminar airflow was not associated with the prevention of PJI in primary TJA, after accounting for confounders and ensuring modern standard-of-care SSI prevention protocols. Contrary to the prior published study from the New Zealand Joint Registry,^15^ the use of LAF was not associated with increased incidence of PJI.

By passing fresh air unidirectionally with a steady velocity and approximately parallel streamlines, the use of LAF creates a zone in which the air, aerosols, and particles within the room are driven out. A previous study revealed that the LAF system results in a reduction of 89% in colony-forming units in comparison with a turbulent ventilation system.^12^ More important, previous studies suggest that the operating room door is opened a mean of 0.65 times per minute during a TJA procedure^3^ and that LAF may be protective against the negative association of the number of people and door openings with air quality inside the operating room.^13^ Despite all these reported benefits, the effectiveness of LAF for decreasing infection after TJA has remained controversial.

Some studies using data from large national registries have demonstrated an increase in infection rates after TJA using LAF, while controlling for potential confounding variables.^15,16,24^ However, those findings should not be interpreted to mean that operating room air quality is unimportant. There are several explanations for the wide variability of reported results with LAF. First, the parallel airflow of the LAF system can be easily disrupted by objects or personnel around the surgical field. Second, the association between air contamination and the deep infection rate is logarithmic. It is necessary to achieve a 10-fold reduction in air contamination to halve the infection rate.^25^ Thus, once a certain level of air quality is achieved, any further reductions in infection rates will be due to the quality of aseptic technique.^26^ Third, LAF systems fail to address the environment outside of the immediate LAF zone, leaving scant room for implant and instrument trays and tables. Laminar airflow systems may be associated with the contamination of these areas by blowing bacteria off personnel and the floor onto instrumentation and other personnel.^27^ Last, the cooling effect of the fresh air from an LAF system on the surgical wound and the patient may be associated with lower intraoperative tissue temperatures in the surgical wound or systemic hypothermia, which may increase the risk of postoperative infection.^28^

Reducing the risk of bacterial contamination during the surgical procedure is a critical strategy for the prevention of PJI after TJA, and adequate intraoperative air treatments remain a critical factor for reducing bacterial contamination. Compliance with best practices within the LAF system has been associated with the potential beneficial effects of LAF. For example, LAF theaters and ultraclean air can only reduce the risk of infection if used in conjunction with strict theater protocol and discipline. However, a questionnaire survey study conducted in the UK reviewed compliance with basic principles of SSI prevention in the operating room where LAF is present and suggested that overreliance on LAF may be associated with slackness in theater protocol adherence where operators believe that the LAF will minimize SSIs without the necessity to comply with best practices.^29^ Furthermore, a Dutch study assessed the positioning of surgical instruments within the clean airflow stream of an LAF system.^30^ The correct positioning of the instrument table (ie, under the LAF canopy) was noted in only 6.1% of 829 procedures, which may be deleterious and result in instrument contamination due to air eddies.

### Limitations

The present study has several limitations. First, it is a retrospective study and therefore may have inherent limitations such as variability in data collection and recording. Second, although we used PSM to minimize the potential effect of confounding variables, we could not control for all variables. Factors such as the number of personnel and amount of traffic inside the operating room, as well as the number of door openings, were not factored into our analysis. However, institutional oversight of the same strict theater protocol and discipline between both of our surgical facilities may help minimize this source of potential bias. Third, this study was conducted at a single institution and the number of cases may be relatively small to examine the posited question, and there may be a potential for type II statistical error. Additional research with larger samples is needed to adequately address this issue. Fourth, this study examined only cases of primary TJA; thus, our conclusion can be applied only to the setting of primary TJA, and not revision arthroplasty, where longer operative times may generate alternative findings.

## Conclusions

The present study supports the recommendation of the International Consensus Meeting on Orthopedic Infections that advocates for clean air in operating rooms that can be accomplished without the use of LAF.^31^ In a cost-conscious health care environment, considering the excessive cost associated with the design and implementation of LAF, it is reasonable to assume that primary TJA can be performed in operating rooms equipped with effective ventilation systems that need not be LAF.
